# Development of an erythropoietin prescription simulator to improve abilities for the prescription of erythropoietin stimulating agents: Is it feasible?

**DOI:** 10.1186/1471-2369-12-11

**Published:** 2011-02-18

**Authors:** Luca Gabutti, Filippo Nobile, Valentina Forni, Fabio Rigamonti, Nadir Weibel, Michel Burnier

**Affiliations:** 1 Division of Nephrology, Ospedale la Carità, Via Ospedale, 6600 Locarno, Switzerland; 2 Department of Internal Medicine, Ospedale la Carità, Locarno, Switzerland; 3 Division of Nephrology, University Hospital of Lausanne, Lausanne, Switzerland; 4 Department of Cognitive Science, University of California, San Diego, USA

## Abstract

**Background:**

The increasing use of erythropoietins with long half-lives and the tendency to lengthen the administration interval to monthly injections call for raising awareness on the pharmacokinetics and risks of new erythropoietin stimulating agents (ESA). Their pharmacodynamic complexity and individual variability limit the possibility of attaining comprehensive clinical experience. In order to help physicians acquiring prescription abilities, we have built a prescription computer model to be used both as a simulator and education tool.

**Methods:**

The pharmacokinetic computer model was developed using Visual Basic on Excel and tested with 3 different ESA half-lives (24, 48 and 138 hours) and 2 administration intervals (weekly vs. monthly). Two groups of 25 nephrologists were exposed to the six randomised combinations of half-life and administration interval. They were asked to achieve and maintain, as precisely as possible, the haemoglobin target of 11-12 g/dL in a simulated naïve patient. Each simulation was repeated twice, with or without randomly generated bleeding episodes.

**Results:**

The simulation using an ESA with a half-life of 138 hours, administered monthly, compared to the other combinations of half-lives and administration intervals, showed an overshooting tendency (percentages of Hb values > 13 g/dL 15.8 ± 18.3 vs. 6.9 ± 12.2; P < 0.01), which was quickly corrected with experience. The prescription ability appeared to be optimal with a 24 hour half-life and weekly administration (ability score indexing values in the target 1.52 ± 0.70 vs. 1.24 ± 0.37; P < 0.05). The monthly prescription interval, as suggested in the literature, was accompanied by less therapeutic adjustments (4.9 ± 2.2 vs. 8.2 ± 4.9; P < 0.001); a direct correlation between haemoglobin variability and number of therapy modifications was found (P < 0.01).

**Conclusions:**

Computer-based simulations can be a useful tool for improving ESA prescription abilities among nephrologists by raising awareness about the pharmacokinetic characteristics of the various ESAs and recognizing the factors that influence haemoglobin variability.

## Background

The recent development of long-acting erythropoietin stimulating agents (ESA), and the clinical trend to increase ESA administration intervals, have markedly changed the ESA prescription profile in nephrology. Thus, from a half-life of 19.4 ± 2.4 hours for erythropoietin alpha [1] and 24.2 ± 2.6 hours for erythropoietin beta [2] after subcutaneous administration, and a recommendation to administer these agents up to three times per week, we are now using compounds such as darbepoetin [3] -and, recently, CERA- that have a much longer half-life (48.8 ± 5.2 hours and 139 ± 20 hours, [4,5] respectively), with the suggestion to lengthen the administration interval up to once monthly. However, the correct application of the new strategies implies that users are well aware of the pharmacological characteristics and risks of the newer long-acting molecules.

Although these assumptions have probably not been met, prescribers seem to have quickly and spontaneously adapted to the new conditions, but numerous observations document important - and sometimes cyclical - fluctuations in the haemoglobin values of chronically-treated patients. This has led to numerous questions, which the current clinical, pharmacokinetic and pharmacodynamic knowledge has been able to answer only in part [6-18]. With respect to haemoglobin cycling in particular, the prescription strategy, especially considering frequent and sudden adjustments in erythropoietin dosage, has been considered a possible triggering factor [19-21], while so far no direct effect of a longer administration interval on haemoglobin stability has been noted. The debate on the causes of haemoglobin variability would be purely academic if the stability of haemoglobin were not to be within a therapeutic margin that, in these last few years, has become narrower, and if the haemoglobin fluctuations had not been associated with a greater morbidity [22].

In order to help physicians acquire prescription ability, and with the hope of reducing haemoglobin variability, we felt it was relevant to build a pharmacokinetic computer model to be used as an ESA prescription simulator. The aim of this simulator is to raise awareness among ESA users about the implications of recent changes in erythropoietin half-life and prescription intervals. For this purpose, we have built a simulator that asks users to prescribe various ESAs for a naïve patient and to adjust the dose as precisely as possible in a 12-week equilibration or balance phase followed by a 20-week maintenance phase (haemoglobin target 11-12 g/dL). The epoetin half-life and the interval of administration will be assigned to the user at the start of the exercise, while the initial haemoglobin and the patient's erythropoietin sensitivity are randomly generated by the software.

Although such a simulator, contrary to other models based, for instance, on Artificial Neural Networks or Bayesian Adaptive Control [23], is not a prediction tool applicable to clinical practice, we feel that it could be a good way to improve the physicians' ability to prescribe ESAs. Moreover, the simulator should enable us to answer the following questions: (1) Is the ability to keep haemoglobin within the target (primary end point) and haemoglobin stability (secondary end point) influenced by the erythropoietin half-life and administration interval and/or by the use of the simulator (learning effect)? (2) Does the number of changes in ESA dosing correlate with the fluctuations in haemoglobin values and administration interval (secondary end point)? (3) Is the intra-patient delta haemoglobin a more sensitive indicator of haemoglobin stability compared to the standard deviation (secondary end point)?

## Methods

### Simulator characteristics

The "epoietin prescription simulator" was developed in Visual Basic on Excel. The version used in the study is annexed to this document (see Additional File 1 named The Epoetin Prescription Simulator). The user's manual as well as the formulae, including a mono-exponential one that relates the erythropoietin half-life selected for the simulation to ESA's concentration and its effect on the production of new red blood cells, are detailed in the Additional File 2 named Appendix 1.

The simulator randomly defines, for an ESA naïve patient, the starting haemoglobin (Hb), with 0.1 g/dL increments, in a range between 7.0 and 8.0 g/dL. To better adjust to clinical practice, it automatically includes, for the duration of the simulation, incidental fluctuations in Hb with an absolute magnitude between -0.5 and +0.5 g/dL [24]. The subject's sensitivity to epoetin is randomly assigned, making sure that the population's average weekly need for epoetin in order to reach the pre-established haemoglobin target of 11-12 g/dL [25] is at approximately 6,000 units. The mean red blood cell (RBC) lifespan in the initial configuration is always set at 61.2 days, and will fluctuate during the simulation according to the erythrocyte age distribution. The amount of weekly epoetin needed, the initial RBC lifespan and the pre-erythrocyte kinetics are simulated according to the data of the literature [26-32].

The home page contains a window with the half-life selected for the test (restricted to 24, 48 and 138 hours), another with the current haemoglobin, an active window where the epoetin dosage can be entered (initially weekly or biweekly and, after 8 weeks, weekly or monthly as predetermined), and finally one showing the statistics of the test in progress from the first week.

Statistics are automatically updated during the simulation and summarise the following parameters: *mean Hb *with *SD*, variability based on the *delta Hb *(average of the difference between consecutive values), *mean RBC lifespan*, percentage of *Hb *values *< 11*, *>12 *and *>13 g/dL*, and a score (*"ability score"*) starting from 1 (meaning that 100% of the values are outside the target range); the score increases with the decrease of Hb values outside the predetermined optimal range of 11-12 g/dL (a haemoglobin value above 13 g/dl is counted as a double error: one point for Hb > 12 and another for Hb > 13 g/dL; see appendix 1 for details). The model also includes the possibility of randomly adding an acute bleeding episode with depletion of blood volume between 0% and 30%.

Considering that the software is annexed to the paper, we remind those users who are outside the current study that, taking into account the simplification of the biological process on which the design was based, and the fact that pharmacodynamic data for erythropoietin are incomplete and affected by significant differences among individuals, the model cannot be used to compare erythropoietin products currently on the market or to prescribe erythropoietin in clinical practice.

### Selected population and procedure

In order to meet the study's needs, we asked two independent groups of 25 nephrologists (both graduates or still pursuing their degree) selected during a national-level meeting to participate by completing the 6 predetermined simulations in random order (erythropoietin with 24, 48 or 138 hours of half-life combined with weekly or monthly administration interval). Each group's participation was planned to be separated by nine months.

The first session was scheduled during CERA's premarketing stage and was designed to be carried out with candidates without experience with the new molecule; the second one was the opposite, taking place after CERA had entered the market. In the first session (simulation A), in order to double the number of equilibration events to which each candidate was subjected, and to analyse the learning curve, an acute bleeding episode (0-30% of blood volume) was randomly introduced into the model between weeks 18 and 24. During the second session (simulation B) we exposed each candidate to the six predetermined simulations but without acute bleeding episodes. In each simulation, candidates were asked to enter an erythropoietin dosage, taking into account the erythropoietin half-life and administration interval already entered with the purpose of reaching as fast as possible and with the maximum precision the Hb target (between 11 and 12 g/dL), adapting the dosage in the following weeks/months as if it was a patient on haemodialysis.

### Statistical analysis

Statistical analyses were performed using a statistical software package (SPSS 12.0; SPSS Inc., Chicago, IL, USA). Results were expressed as mean ± SD. Intra-patient [33] haemoglobin variability other than SD was estimated from the average of the absolute value of the differences between consecutive parameters defined in the text "delta Hb". The haemoglobin target was selected with a narrow margin (11-12 g/dL) to improve the likelihood of finding differences between the groups. Comparisons between parameters were carried out with a paired t-test, while Hb profiles as a function of time were compared using a trapezoidal estimation of the area under the curves followed by a Wilcoxon Signed Ranks test. Percentages were compared using a Fisher Exact test. In all cases, a *P *≤ 0.05 was considered statistically significant; *P *was expressed as *ns *(not significant), 0.05, <0.05, <0.01 and <0.001.

## Results

**Figure 1:**
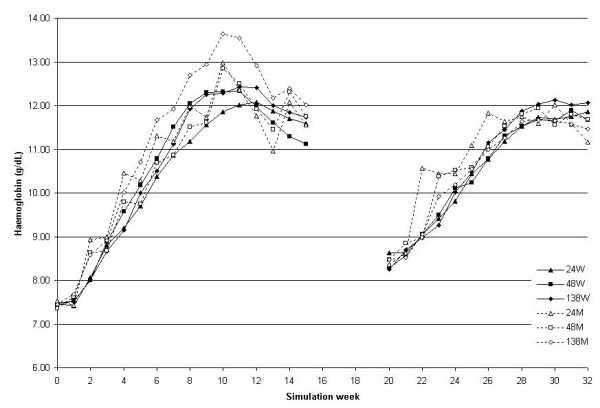
**Haemoglobin course; simulation A (before CERA's marketing)**. Hb as a function of the weeks elapsed since the start of the simulation for the 6 combinations of half-lives and administration intervals (half-life in h: 24, 48 and 138; administration interval, weekly (W) or monthly (M); 24W solid line with black triangles, 48W solid line with black squares, 138W solid line with black diamonds, 24M dotted line with white triangles, 48M dotted line with white squares, 138M dotted line with white diamonds). The randomly-assigned bleeding phase between the two equilibration exercises is not represented; the second equilibration phase is synchronised. N = 25.

The Hb course as a function of time (expressed as simulation weeks) and the average Hb in each simulation modality are shown in Figure 1 and Table 1, respectively. In order to facilitate the comparison of the different curves, the bleeding phases are not represented in the graph and the curves have been synchronised. Thus, the second equilibration phase starts on the graph in the same week for each patient and each modality. As can be observed, and even if the curves with the exception of 138M were not statistically different, the combination of half-life and administration interval that, in the equilibration phase, mostly respected the target range was 24 hours with weekly administration (24W). The only curve on Figure 1 that was significantly different from the others (P < 0.01) was associated with overshooting: the 138M (138 h half-life and monthly administration interval). With this combination, and comparing with the other simulations, the percentage of Hb values > 13 g/dL was 15.8 ± 18.3% vs. 6.9 ± 12.2%; P < 0.01). To support a favourable learning effect, no significant differences between groups were observed during the equilibration phase following the bleeding episode. In this regard, comparing with the first equilibration phase, the overall dispersion of Hb values outside the target range was significantly smaller: average Hb dispersion in the last 8 weeks of the 2 equilibration phases 1.32 ± 0.28 vs. 0.63 ± 0.26 g/dL; P < 0.01; Hb percentage values outside the target of 11-12 g/dL 50.0 ± 30.9 vs. 18.8 ± 28.8%; P = 0.05) (secondary end-point). Haemoglobin variability is detailed in Table 1 using as parameters the SD and the average of the absolute value of the difference between consecutive measurements "delta Hb".

**Table 1:** Haemoglobin variability in simulation A

	**Mean Hb (g/dL)**	**SD**	**Delta Hb**
	
**24W**	10.37	1.65	1.19
**24M**	10.55	1.59	1.26
**48W**	10.41	1.68	1.28
**48M**	10.53	1.66	1.17
**138W**	10.55	1.86	1.34
**138M**	10.76	1.88	1.39

Average Hb and variability expressed as standard deviation and delta Hb (average of the absolute value of the difference between consecutive measurements) for the 6 modalities. Half-life in h: 24, 48 and 138; administration interval, weekly (W) or monthly (M). N = 25

**Figure 2:**
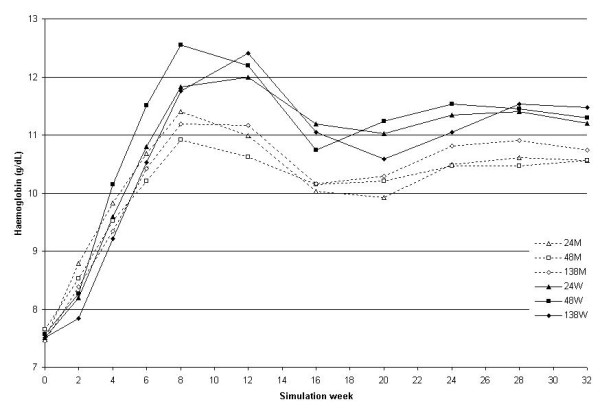
**Haemoglobin course in simulation B (after CERA's marketing). **Hb as a function of the weeks elapsed since the start of the simulation for the 6 combinations of half-lives and administration intervals (half-life in h: 24, 48 and 138; administration interval, weekly (W) or monthly (M); 24W solid line with black triangles, 48W solid line with black squares, 138W solid line with black diamonds, 24M dotted line with white triangles, 48M dotted line with white squares, 138M dotted line with white diamonds). To better evaluate the equilibration phase no bleeding episodes were introduced. N = 25.

Changes in Hb observed during the second simulation (simulation B), which was carried out after the marketing of CERA are shown in Figure 2. As shown in the Figure, no curves were characterized by an evident overshooting of (mean Hb > 13g/dL).

**Figure 3:**
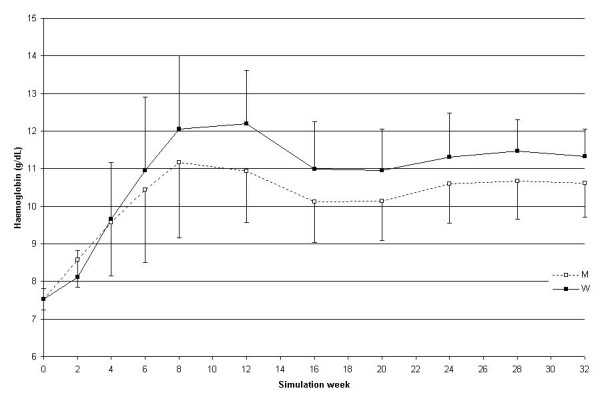
**Monthly haemoglobin course comparing monthly and weekly administration intervals; simulation B. **Monthly Hb as a function of the weeks elapsed since the start of the simulation for the 2 administration intervals: monthly M (dotted line with white squares) or weekly W (solid line with black squares). N = 25

**Figure 4:**
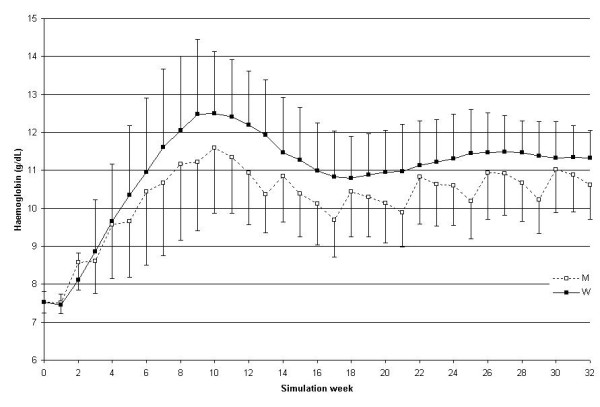
**Weekly haemoglobin course comparing monthly and weekly administration intervals; simulation B. **Weekly Hb as a function of the weeks elapsed since the start of the simulation for the 2 administration intervals: monthly M (dotted line with black squares) or weekly W (solid line with black squares). The simulator user was aware of the monthly values only. N = 25.

The comparison between monthly and weekly administration is shown in Figure 3 (where only Hb values that were visible to the simulator user are represented) and Figure 4 (representing the weekly haemoglobin values for the entire simulation).

**Figure 5:**
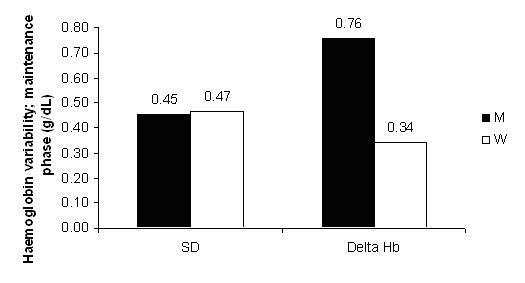
**Haemoglobin variability in simulation B. **Hb variability expressed as standard deviation (SD) and delta Hb (absolute value of the difference between consecutive measurements) comparing the 2 administration intervals: monthly M (black columns) and weekly W (white columns). The difference between columns in "Delta Hb" is significant; P < 0.01. N = 25.

Haemoglobin variability during the maintenance phase is represented in Figure 5, using the SD and the average of the absolute value of the difference "delta Hb" between consecutive measurements. The monthly administration interval compared with the weekly one was associated with a significantly higher delta Hb (0.76 vs. 0.34 g/dL; P < 0.01). The SD was not able to detect the difference (secondary end-point). The same parameters are shown in Table 2 for the entire simulation.

**Table 2:** Haemoglobin values and ability score; simulation B

	**24M**	**24W**	**48M**	**48W**	**138M**	**138W**	**M**	**W**	***P***
	
**Haemoglobin (g/dL) Mean**	10.38	11.10	10.20	11.36	10.32	10.82	**10.30**	**11.09**	<001
***SD***	1.23	1.57	1.15	1.91	1.33	1.62	1.24	1.70	
**Delta Hb**	0.82	1.01	0.70	1.33	0.85	0.98	0.79	1.11	
**Haemoglobin values <11 g/dL (%)**	70.39	44.22	73.28	45.94	65.73	53.58	**69.80**	**47.91**	<0.001
***SD***	26.57	23.48	25.50	20.92	27.92	21.17	26.66	21.85	
**Haemoglobin values >12 g/dL (%)**	12.53	23.68	8.68	30.18	14.32	21.90	**11.84**	**25.25**	<0.01
***SD***	17.38	24.36	14.65	20.73	20.58	19.10	17.54	21.39	
**Haemoglobin values >13 g/dL (%)**	5.51	10.77	2.89	17.09	4.60	11.02	**4.33**	**12.69**	<0.01
***SD***	11.89	16.80	7.74	18.64	9.74	13.41	9.79	16.28	
**Ability score**	1.20	1.52	1.22	1.23	1.25	1.24	1.22	1.33	ns
***SD***	0.33	0.70	0.23	0.57	0.31	0.34	0.29	0.54	

Mean haemoglobin with SD and delta Hb, value percentages outside the target (during the 32 weeks of the simulation) and ability score for the 6 modalities, comparing monthly to weekly administration (half-life of 24, 48 and 138 hours; monthly M and weekly W administration) (simulation B). N = 25

Table 2 shows the percentage of values outside the target during the 32 weeks of the simulation for each modality, comparing monthly to weekly administration. Monthly administration is characterised by a lower percentage of Hb values at the target of 11-12 g/dL (18.4 vs. 26.8%; P < 0.05), but there are also fewer values above 12 or 13 g/dL (11.8 vs. 25.3%; P < 0.01 and 4.3 vs. 13.0; P < 0.05). With respect to the ability score, the only modality that stands out from the rest in a significant way is that associating the short half-life (24 hours) with the weekly administration (1.52 ± 0.70 vs. 1.24 ± 0.37; P < 0.05) (primary end point).

**Figure 6:**
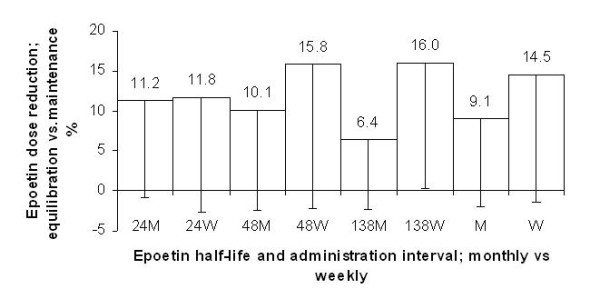
**Epoetin dose reduction, equilibration vs. maintenance in simulation B. **Error in determining the maintenance dose estimated by calculating the percentage difference between the mean erythropoietin dose used in the equilibration phase and that used in the maintenance phase (half-life of 24, 48 and 138 h; monthly M and weekly W administration). N = 25.

The error in determining the maintenance dose was estimated by calculating the difference between the mean weekly dose of erythropoietin used in the equilibration phase and that used in the maintenance phase (6084 ± 3057 vs. 5575 ± 1828 U/Week). The discrepancy, as illustrated in Figure 6, was found to be larger for weekly than for monthly administration (14.5 ± 15.9 vs. 9.1 ± 11.0%; P < 0.05), with differences increasing together with the half-life of the ESA.

**Figure 7:**
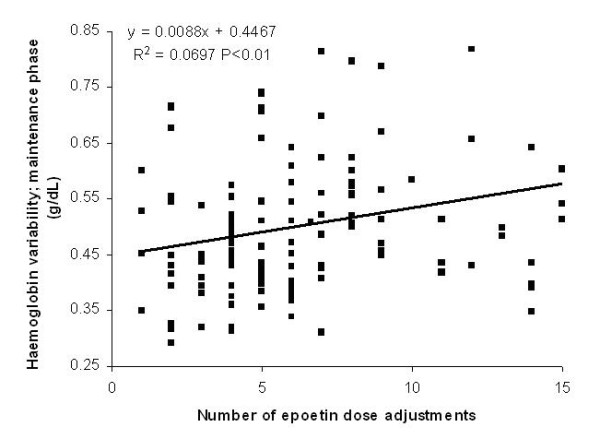
**Haemoglobin variability and epoetin dose adjustments in simulation B. **Correlation between the number of therapy modifications and Hb variability (expressed as delta Hb) in the maintenance phase. N = 25.

Whatever the drug half-life, the weekly administration of ESA was associated with a significantly higher number of adjustments of the doses (4.9 ± 2.2 vs. 8.2 ± 4.9; P < 0.001). Figure 7 presents the significant direct correlation between the number of therapy modifications and haemoglobin variability, expressed as delta Hb, during the maintenance phase (secondary end-point).

**Figure 8:**
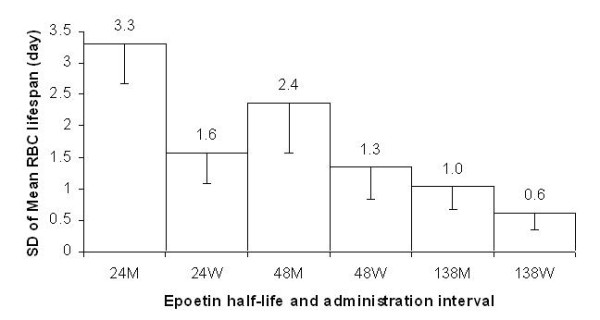
**RBC lifespan variability in simulation B. **RBC lifespan variability (expressed as standard deviation) as a function of erythropoietin half-life and administration interval (half-life in hours, 24, 48 and 138; administration interval, weekly W or monthly M). N = 25.

Interestingly, both the half-life of erythropoietin and the administration interval appear to affect the age distribution of red blood cells (RBC) within the circulating population, modifying the mean RBC lifespan. The variability of the RBC lifespan is expressed in Figure 8 as SD. The variability decreases with an increase in the half-life of erythropoietin and with a decrease in the administration interval.

## Discussion

The purpose of this study was to develop a new tool to improve the physicians' ability to prescribe erythropoietin stimulating agents in dialysis patients and hence to raise awareness on the pharmacological consequences resulting from the use of ESAs with a very long half-life over longer administration intervals. Our two simulations (A and B), performed before and after CERA's marketing, enable us to conclude that the simulator is user-friendly and that its use is associated with learning (less dispersion of Hb values and improvement of the ability to respect the predetermined target).

Simulation modality A was tested with a group of nephrologists who had never used erythropoietins with half-lives longer than 48 hours, showing that the first approach with a prolonged half-life (138 hours) and a monthly administration interval could have overshooting as a consequence. Nevertheless, the risk of overshooting is quickly corrected thanks to the learning, and the prescriber is already able to avoid the administration of an excessive dose in the second equilibration phase of the same simulation.

Interestingly, in simulation B, performed after CERA's marketing with a group of nephrologists being aware of its long half-life (about 139 hours), overshooting tendencies were not observed. The same simulation, compared with the first one, allowed a more detailed analysis of the maintenance phase following the initial equilibration phase. Surprisingly, the simulation has shown that nephrologists have been more careful and conservative with prescriptions when faced with an administration interval of 4 weeks. The result was that monthly administration has been translated into a significantly lower average Hb value (10.3 vs. 11.1 g/dL; P < 0.01), as well as a lower percentage of Hb values at the target of 11-12 g/dL (18.4 vs. 26.8%; P < 0.05). Accordingly, with the type of ESA and the administration interval mostly used at the time the study was carried out, the only modality that was characterised with a prescription ability (calculated by indexing the percentage of parameter values on target, below target, above target, and above the safety Hb value of 13 g/dL) that was statistically superior compared to the others (primary end point) was the combination of the shortest half-life (24 hours) with weekly administration (ability score 1.52 vs. 1.24; P < 0.05). The smaller adjustments needed, regarding the mean ESA dose, between the equilibration and maintenance phases using the monthly administration interval (9.1 vs. 14.5%; P < 0.05) was probably due to the lower mean Hb value at the end of the equilibration phase when the monthly interval was used; so it should not be interpreted as better prescription ability but as more prudence when faced with long half-lives.

As can be observed when comparing Figures 2 and 3, the weekly Hb value shows larger variability when using monthly compared to weekly administration (only the monthly values as shown in Figure 2 were visible to the prescriber). The greatest variability in the model is generated by red blood cell sub-populations of different ages (a fact shown by the curve behaviour in Figure 3 as well as by the significant difference in the fluctuation of red blood cell lifespan during the simulation summarised in Figure 8), caused by the single monthly dose of erythropoietin that has resulted in a non-homogeneous generation and elimination of red blood cells over time.

Hb variability analysis enables a critical evaluation of the meaning of standard deviation (average distance of individual values from the mean) compared to delta Hb (average of the absolute value of the difference between consecutive measurements). In the particular case of the simulation, delta Hb is in fact the parameter that best translates incidental changes in Hb values.

Using a monthly instead of a weekly administration interval, the number of modifications to the erythropoietin dose is significantly lower (4.9 vs. 8.2; P < 0.001). As suggested in the literature [19] and demonstrated with the significant direct correlation between Hb variability and number of epoetin dose adjustements (Figure 7), this fact could have favourable consequences on Hb stability.

## Conclusions

In conclusion, bearing in mind the limitations of our model, the results obtained with our simulator could be used as a path for further experimental studies. An ESA prescription simulator can be a useful educational tool to raise awareness about the possible consequences of changing the medication's half-life or its administration interval. The first time users were faced with an erythropoietin compound with a half-life of 138 hours and a monthly administration interval, they were exposed to a risk of overshooting, which was corrected by the training on the simulator. With respect to possible consequences, very long half-lives and monthly administration intervals translate into a conservative prescription that maintains Hb values below the desired level. As a consequence the shortest half-life (24 hours) with the weekly administration interval is associated with the better prescription ability. The monthly prescription interval however, as suggested in the literature, is accompanied by less therapeutic adjustments; in this regard a direct correlation between Hb variability and number of therapy modifications has been demonstrated. In our simulations, the delta Hb has proven to be a better tool for evaluating intra-patient Hb variability than standard deviation.

Computer-based simulation tools can be particularly useful for improving prescription patterns and for testing new working hypotheses such as determining the factors that influence Hb variability. Additional knowledge on the pharmacodynamic of ESAs is necessary to fine-tune the models and bring them closer to the level of regular clinical experience. However, the research of possible consequences of half-life and administration interval for ESAs that are currently marketed with respect to the Hb stability will require specific targeted clinical trials.

## Competing interests

The authors declare that they have no competing interests.

## Authors' contributions

LG was involved in the study design, sample collection, analysis and interpretation of the data, in building the simulation model and in writing the report; FN and VF participated in the sample collection, analysis and interpretation of the data and in writing the paper; FR and WN participated in building the simulation model, MB helped formulate the study design, the data analysis strategy and contributed to writing the paper. All authors have read and approved the final version of the manuscript.

## Pre-publication history

The pre-publication history for this paper can be accessed here:

http://www.biomedcentral.com/1471-2369/12/11/prepub

## Supplementary Material

Additional File 1 **The epoetin prescription simulator**. Visual Basic version on Excel of the pharmacokinetic simulation tool used in the study. Click here for file

Additional File 2 **Appendix 1**. Characteristics of the "Epoetin Prescription Simulator" tool and user's manual. Click here for file
