# Supplementary material for: Development of an erythropoietin prescription simulator to improve abilities for the prescription of erythropoietin stimulating agents: Is it feasible?
Source: BMC Nephrol. 2011 Feb 18;12:11. doi: 10.1186/1471-2369-12-11 (PMC3055807; doi:10.1186/1471-2369-12-11)
Supplement: Additional File 2 — Appendix 1. Characteristics of the "Epoetin Prescription Simulator" tool and user's manual. [file 1471-2369-12-11-S2.DOCX]

***Appendix 1***

**Characteristics of the “Epoetin Prescription Simulator” and user’s manual:**

The “epoetin prescription simulator” has been developed in Visual Basic on Excel. The version used in the study is annexed to the manuscript, and can be activated and used in Excel without the need for links (see **attached file** named **“The epoetin prescription simulator; BMC nephrology”**).

Once activated, to go from the initial sheet that provides graphical support to the simulator (called “simulation”) to the one that contains the software proper (called “analysis”), press the “show analysis” button and enter 12345 for the password.

The formulae, including a mono-exponential one that relates the erythropoietin half-life selected for the simulation to ESA’s concentration at any time and to its effect on the production of new red blood cells are listed at the bottom of the appendix and can be visualised in the analysis sheet by selecting the “unprotect sheets” command.

The simulator randomly defines, for an ESA naïve patient, the starting haemoglobin (Hb), with 0.1 g/dL increments, in a range between 7.0 and 8.0 g/dL and, to better adjust to clinical practice, automatically includes - for the duration of the simulation - incidental fluctuations in Hb with an absolute magnitude between -0.5 and +0.5 g/dL [24]. The subject’s sensitivity to epoetin is assigned from a factor of 1 (very sensitive) to a factor of 7 (hardly sensitive), making sure that the average weekly need for epoetin of the population in order to reach the pre-established haemoglobin target of 11-12 g/dL [25] is at approximately 6,000 units. The mean red blood cell (RBC) lifespan in the initial configuration is always set at 61.2 days and fluctuates during the simulation according to the homogeneous or inhomogeneous distribution of the erythrocyte age. The amount of weekly epoetin needed, the initial RBC lifespan and the pre-erythrocyte kinetic are simulated according to the data of the literature [26-32].

In the “analysis” sheet, activating the “Reset” option generates a new case; this process is automatically executed each time the simulator is opened.

The home page contains a small welcome and introduction that briefly describes the aims of the study, a window that records the half-life of the erythropoietin currently on the Swiss market, the half-life selected for the test (“Selected epoetin half-life”) - which can be modified without any restriction on the “analysis” sheet and was restricted to 24, 48 and 138 hours in the present study, a window with the current haemoglobin, an active window where the epoietin dosage can be entered (initially weekly or biweekly and, after 8 weeks, weekly or monthly as predetermined in the “analysis” sheet), and a window showing from the first week the statistics of the test in progress.

Statistics of the Hb course are automatically updated during the simulation and summarise the following parameters over the 32-week period: *mean Hb* with *SD*, variability based on the *delta Hb* (average of the difference between consecutive values), *mean RBC lifespan*, percentage of *Hb* values *< 11*, *>12* and *>13 g/dL*, and a score *“ability score”* that starting from 1 (meaning that 100% of the values are outside the target range) increases with the decrease of values outside the predetermined optimal range of 11-12 g/dL (an haemoglobin value above 13 g/dl is counted as a double error: one point for Hb>12 and another for Hb>13 g/dL; see the formula at the bottom of the appendix for details). The model also includes the possibility of randomly adding an acute bleeding episode with depletion of blood volume between 0% and 30%, or an acute transient decrease in the sensitivity to epoietin to simulate an acute inflammatory process (this last option has not been used in the study).

The model was developed in collaboration with an engineer from the Department of Cognitive Science of the University of California, San Diego, USA.

**Warning:**

We remind the users of the software who are outside the current study that, taking into account the simplification of the biological process on which the design was based, and the fact that pharmacodynamic data for erythropoietin are incomplete and affected by significant differences among individuals, the model cannot be used to compare erythropoietin products currently on the market or to prescribe erythropoietin in clinical practice.

**Formulae:**

1. ESA available on day **x** in units = ESA administered on day **x** in units* +

(ESA administered on day **x**-7 in units* / 2 ^(hours since the last administration/ESA’s half-life in hours)^ )

*corrected by the ESA’s half-life to obtain the same area under the curve dose/responsiveness

1. Reticulocytes generation on day **x** = basis reticulocytes generation^#^ + (ESA available on day **x** of the current week in units/ 2 ^(hours elapsed since the beginning of the week/ ESA’s half-life in hours)^ / incidental EPO sensitivity / 100 )

^#^calculated to obtain a basis Hb between 7.0 and 8.0 g/dL

1. RBC count on day **x** of the week y = RBC count on the day **x** of the week **y**-1 – (RBC count on day **x** of the week **y**-1) . ( incidental bleeding % on day **x** )
2. Ability score = 100 / % of Hb values < 11g/dL^&^ + % of Hb values > 12 g/dL^&^ + % of Hb values >13 g/dL^&^

^&^during the whole simulation
